# Costing analysis of an SMS-based intervention to promote HIV self-testing amongst truckers and sex workers in Kenya

**DOI:** 10.1371/journal.pone.0197305

**Published:** 2018-07-06

**Authors:** Gavin George, Taruna Chetty, Michael Strauss, Silas Inoti, Samuel Kinyanjui, Eva Mwai, Matthew L. Romo, Faith Oruko, Jacob O. Odhiambo, Eston Nyaga, Joanne E. Mantell, Kaymarlin Govender, Elizabeth A. Kelvin

**Affiliations:** 1 Health Economics and HIV and AIDS Research Division, University of KwaZulu-Natal, Durban, South Africa; 2 North Star Alliance, Nairobi, Kenya; 3 Department of Epidemiology and Biostatistics, CUNY Graduate School of Public Health and Health Policy, City University of New York, New York, United States of America; 4 Institute for Implementation Science in Population Health, City University of New York, New York, United States of America; 5 HIV Center for Clinical and Behavioral Studies, Department of Psychiatry, Division of Gender, Sexuality and Health, New York State Psychiatric Institute & Columbia University, New York, New York, United States of America; University of Queensland, AUSTRALIA

## Abstract

**Objective:**

HIV testing rates in many sub-Saharan African countries have remained suboptimal, and there is an urgent need to explore strategic yet cost-effective approaches to increase the uptake of HIV testing, especially among high-risk populations.

**Methods:**

A costing analysis was conducted for a randomized controlled trial (RCT) with male truckers and female sex workers (FSWs) registered in the electronic health record system (EHRS) of the North Star Alliance, which offers healthcare services at major transit hubs in Southern and East Africa. The RCT selected a sample of truckers and FSWs who were irregular HIV testers, according to the EHRS, and evaluated the effect of SMSs promoting the availability of HIV self-testing (HIVST) kits in Kenyan clinics (intervention program) versus a general SMS reminding clients to test for HIV (enhanced and standard program) on HIV testing rates. In this paper, we calculated costs from a provider perspective using a mixed-methods approach to identify, measure, and value the resources utilized within the intervention and standard programs. The results of the analysis reflect the cost per client tested.

**Results:**

The cost of offering HIVST was calculated to be double that of routine facility-based testing (USD 10.13 versus USD 5.01 per client tested), primarily due to the high price of the self-test kit. In the two study arms that only offered provider-administered HIV testing in the clinic, only 1% of truckers and 6% of FSWs tested during the study period, while in the intervention arm, which also offered HST, approximately 4% of truckers and 11% of FSWs tested. These lower than expected outcomes resulted in relatively high cost per client estimates for all three study arms. Within the intervention arm, 65% of truckers and 72% of FSWs who tested chose the HIVST option. However, within the intervention arm, the cost per additional client tested was lower for FSWs than for truckers, at USD 0.15 per additional client tested versus USD 0.58 per additional client tested, driven primarily by the higher response rates.

**Conclusion:**

Whilst the availability of HIVST increased HIV testing among both truckers and FSWs, the cost of providing HIVST is higher than that of a routine health facility-based test, driven primarily by the price of the HIV self-test kit. Future research needs to identify strategies which increase demand for HIVST, and determine whether these strategies and the subsequent increased demand for HIVST are cost-effective in relation to the conventional facility based testing currently available.

## Introduction

HIV counselling and testing (HCT) is a key component of the HIV response to achieve the UNAIDS Fast Track goals [1]. Increasing the availability of HIV self-testing (HIVST) is recommended as a way of increasing coverage, especially among hard-to-reach and high-risk populations [1], such as long distance truckers and female sex workers (FSWs) in Africa [2–8]. A number of risk factors drive the high prevalence rates amongst truckers, including engagement with FSWs [9], while both truckers and FSWs have been shown to engage in multiple concurrent sexual partnerships [10–12] and use condoms inconsistently [4]. Additionally, studies have revealed that truck drivers and FSWs have inadequate access to health services [5, 9, 13, 14]. Although health clinics are positioned along many major trucking routes in Africa, studies indicate that the uptake of HIV testing amongst these populations remains low [5, 15].

HIVST is considered a safe and effective strategy for increasing the uptake of HIV testing among high-risk and hard-to-reach populations [12, 16, 17]. HIVST was found to be a highly acceptable testing option among FSWs and truckers [15, 18], including in Kenya [19]. In 2009, Kenya became the first African country to develop guidelines on HIVST, and promote the use of oral self-test kits [20]. However, interventions are required to promote HIVST with the aim of maximising uptake, especially among high risk populations not testing regularly with the current standard testing options, and ensuring the cost-effectiveness and efficiency of this strategy [21]. Furthermore, mHealth interventions utilising mobile technology for health promotion have been recommended as a low-cost strategy to increase the uptake of HIV testing, with the potential for broad coverage [22–24].

In this paper we present a costing analysis conducted on a randomized controlled trial (RCT) evaluating the impact of SMSs announcing the availability of HIVST versus a general SMS reminder to test for HIV on HIV testing rates in the eight North Star Alliance clinics in Kenya. The study population comprised male truckers and FSWs registered as clients in the North Star Alliance electronic health record system (EHRS), and who were listed as irregular HIV testers according to the EHRS.

## Methods

### Study setting

The North Star Alliance provides health services to hard-to-reach populations across Africa, including truckers and FSWs. In 2017, the North Star Alliance operated 53 clinics located at major transit hubs in 13 countries in Southern and East Africa, including eight in Kenya where we offered HIVST as an HIV testing option to selected clients. The clinics are open at hours suitable to the target populations and offer a range of prevention and treatment services, including infectious disease (STI, HIV, TB, malaria) screening and treatment, diagnosis and treatment of mobility-related and other non-communicable diseases, health education and, in some clinics, laboratory services.

### Sample, eligibility and consent

For this study we capitalized on the EHRS used by the North Star clinics and selected all truckers and FSWs registered in the system who met the following eligibility criteria: (a) no indication that they were HIV+; (b) reside in Kenya; (c) had a valid mobile phone number listed; (d) tested for HIV fewer than four times in the past 12 months (indicating that they were not following the recommendation to test every 3 months per year); (e) had not tested for HIV in the past three months; (f) had not participated in our previous study on self-administered HIVST; (g) had an indication of male sex in the system, and were listed as working as truck drivers or trucking assistants (turn boys) or of female sex and were listed as working as a sex worker.

For this study, we used a passive consent process due to the large number of participants, making obtaining consent from each individual unfeasible, and the low risk associated with the intervention. The North Star Alliance sent the following consent text message to all eligible clients twice, once in Kiswahili and once in English, a week apart.

“North Star Alliance is evaluating our programs for their improvement using client information from our system. The information we use for this evaluation will not be linked to your name and you will not be contacted or have any expenses related to your inclusion. If you have questions about the use of your data, call [phone number of clinic where they had last been seen]. To have your data excluded, reply “NO” to this text”.

After each consent message, any clients who contacted us indicating they wanted to opt out of having their data included were removed from the sample prior to randomization. A total of 27 truckers and 138 FSWs contacted us to have their data excluded prior to randomization and 2 truckers contacted us after randomization and were considered study drop-outs and their data were removed before analyses. This left a total of 2262 truckers and 2196 FSWs who tacitly agreed to participate in the study and who were subsequently randomized to one of three study arms.

### Description of study arms

The study was conducted between December 2016 and April 2017. Truckers were sampled initially, in December 2016, followed by FSWs in February 2017, and sent the consent SMS messages. After the passive consenting process, study SMSs were sent over a three-week period starting in December 2016 for truckers and March 2017 for FSWs. The follow-up period ensued for the two months following the first study SMSs message sent to the standard of care (SOC), Enhanced SOC and Intervention participants, at which point the data was downloaded from the EHRS for analysis. The three study arms of the RCT are described in Table 1.

**Table 1:** Description of study arms.

**Standard of Care (N = 762 truckers and 696 FSWs)**	Participants received a SMS stating, “North Star Alliance East Africa would wish to kindly remind you to visit any of our Roadside Wellness Centres for HIV testing. Your health, our priority.” This message was sent only once in both English and Kiswahili at the beginning of the study period. Those in this study arm who came to any North Star Alliance clinic were offered only the standard provider-administered blood-based HIV test, which is offered to all North Star Alliance clinic clients.
**Enhanced SOC** (N = 748 truckers and 750 FSWs)	Participants received the SOC SMS described above, sent once a week for three weeks, twice in English and once in Kiswahili. Those in this study arm who came to any North Star Alliance clinic were offered only the SOC test.
**Intervention** (N = 750 truckers and 750 FSWs)	Participants received a SMS promoting the availability of oral self-administered HIVST kits in all 8 North Star Alliance clinics in Kenya, for either home or clinic use. This message was sent once a week for three weeks, twice in English and once in Kiswahili. The message read: “You can now self-test at home or in the clinic for HIV using a new test kit available from all North Star Alliance clinics in Kenya. Your health, our priority.” Participants who came to a North Star Alliance clinic in Kenya were given a brief demonstration of the HIVST kit and then offered a choice of (1) the SOC test; (2) the self-administered oral HIVST for use in the clinic with provider supervision; or (3) a self-administered oral HIVST kit for home use with telephonic post-test counselling.

A clinic receptionist determined which study arm presenting clients belonged to by looking-up the client’s mobile phone number on a spreadsheet listing the numbers of study participants in the intervention arm. The counsellor was then informed when an Intervention client presented so s/he would be given a demonstration of the HIVST kit and then offered the testing choices. Those in the study who visited a North Star Alliance clinic outside of Kenya would be offered the SOC test only as those clinics did not have HIVST kits.

In addition, if someone not in the intervention arm came to a Kenyan clinic and specifically requested a HIVST kit, presumably having heard about them from someone in the Intervention arm, they were given a HIVST kit so as not to lose an HIV testing opportunity.

### HIV testing procedures

Those who tested in the clinic (the SOC test or HIVST with supervision) underwent the standard pre- and post-testing counselling process. Additionally, those who chose HIVST for supervised use in the clinic were given the OraQuick HIVST kit (OraSure Technologies, 2017) with written (English and Kiswahili) and pictorial instructions in a private room. An HCT counsellor sat in the room with the study participant while s/he used the HIV test kit (supervised self-administration) in order to answer any questions that arose during the test administration and to provide guidance if required. Upon the availability of the HIV test result 20 minutes later, the client was given the option to view the results in private or with the counsellor. After viewing the HIV test results, the client received the standard post-test counselling and any referrals if appropriate. If the client chose to view the test results in private, s/he was encouraged to disclose the test results during post-test counselling, but the final decision whether or not to disclose was the clients’. If s/he did not disclose the results, the counsellor proceeded with post-test counselling regardless of whether the HIV test result was positive or negative, including information about accessing HIV care, in the event the test result was positive.

Those who chose to take a HIVST kit for use outside of the clinic were pre-test counselled in the clinic, instructed to use their test within three days, and call or send a SMS after using the test to receive a call-back for post-test counselling and any referrals if appropriate. Participants who failed to contact the clinic staff within the three days after taking a test kit were called to inquire about the use of the test and provide counselling and referrals if appropriate. Clients were informed that they could call or send a SMS at any time while self-testing if they had any questions or concerns. As with in-clinic self-testing, clients were encouraged to disclose their test result during post-test counselling, but this remained the client’s choice, and if s/he did not disclose, the counsellor provided the information for both HIV test outcome scenarios.

### Costing perspective and approach

Costs were estimated from the provider perspective using 2016 prices. Costs incurred prior to this period were adjusted for price year differences using the Campbell and Cochrane Economics Methods Group (CCEMG) and the Evidence for Policy and Practice Information and Coordinating Centre (EPPI-Centre) Cost Converter [25]. Costs were estimated in Kenyan Shillings (KES), and in cases where costs were obtained in other international currencies, they were converted into the local currency [26]. Once the analysis was complete, the results were converted to US dollars (USD) for international comparative purposes. The study used an average annual exchange rate for the base year (2016) of 101.51 KES/ 1 USD [27]. The unit of analysis identified by this study is the HCT client; hence, this costing analysis quantified the cost per client tested.

A mixed-methods approach was used to identify, measure and value costs [28]. Costs were identified and valued using a bottom-up micro-costing approach where data were available, with the remainder estimated using a top-down gross-costing approach [28]. Resources were allocated in line with an activity-based approach [26, 29] according to their economic classification, as per the activities identified:

1. Sending SMSs
2. Pre-counselling
3. HIV Testing
4. Post-test counselling
5. Call-back counselling

A secondary classification between direct and indirect costs was then established [28]. In the context of this study, direct costs arose solely from the provision of the HCT service and were allocated as such. Direct costs emanating from this study included staff directly involved in HCT, as well as consumables used during the HCT process. All costs related to the provision of the SMS service were assigned to that particular activity. All indirect costs identified (those not specifically borne by the HCT service) were allocated proportionally. All resources with a lifespan greater than a year were treated as capital items and costed as such [26]. In addition to infrastructure and equipment, this included non-recurrent training and recruitment costs.

### Data collection and analysis

The cost of providing HIV testing to clients in the different study arms depended on a number of key economic inputs, including staff, consumables and medical supplies, equipment and infrastructure, training, and facility management and supervision. The approach to the collection and analysis of the data is described in the following sections:

1. Measurement and valuation of cost items
2. Cost allocation
3. Cost and outcomes comparison
4. Sensitivity analysis

1. Measurement and valuation of cost items

Per client costs were estimated by activity for each resource identified. For certain resources, unit costs were available at the client level, which were multiplied by the patient utilization of that resource to obtain a cost per client estimate [28]. In the absence of client-level data, average monthly costs per clinic were divided by the average number of HCT clients per month utilizing the clinic, in order to arrive at a cost per client estimate [28].

Patient utilization data were collected using questionnaires designed to first identify the exhaustive list of resources used within each of the activities, and second, to estimate the patient utilization of each resource–the cost-identification process for each activity [26, 28].

Patient utilization questionnaires were distributed to clinic staff involved in HCT-related functions, as well as relevant head office staff directly involved with the management of the facilities. All data were verified in interviews held with relevant head office staff or facility managers. The questionnaires became more specific and detailed with each round of data collection until the integrity and level of detail of the patient utilization data was satisfactory [26].

To value each resource used, interviews were held with relevant head office staff as well as facility managers to obtain cost information [26, 28]. As with the patient utilization data collection process, questionnaires became more specific and detailed with each round of data collection until the integrity and detail of unit cost data was satisfactory. Where costs and costing information could not be ascertained, assumptions were made and rationalized.

Capital costs, such as infrastructure, furniture and equipment, incurred in all three study arms were annuitized and apportioned according to testing uptake [26, 28]. The annuity calculation was performed in Microsoft Excel 2016 using the PMT function and required inputs such as discount rate, lifespan of the item, and purchase price. These inputs were converted to monthly values in order to estimate the replacement value for each of the capital items. This study used a discount rate of 3%, as employed in other costing studies reviewed [17, 30, 31]. Where possible, estimates of useful life were used that were specific to the resource [26, 28], instead of annuitizing all capital inputs over a three- to five-year period like other costing studies [17, 30, 31]. This was considered to be a more contextually-relevant approach [28].

2. Cost allocation

Indirect costs and those shared across all North Star Alliance facilities were allocated to the HCT function according to the proportion of clients utilizing HCT services at the facilities, estimated to be 44.41% [17, 28, 30]. This was verified by the facility managers who estimated that the HCT function accounts for almost half of the clinic’s resources and staff time.

The counsellors reported spending 30 minutes per client on average, for HCT. More specifically, for the average HCT client (in the SOC or Enhanced SOC arms), the 30-minute period was utilized as follows: 5 minutes was spent on pre-counselling (Activity B), 20 minutes conducting the HIV test (Activity C), and 5 minutes on post-test counselling (Activity D). Thus, the HCT share of indirect or shared costs was apportioned to each activity within the HCT function [29] accordingly: Activities B (5/30), C (20/30) and D (5/30), based on the fraction of time spent by the counsellors on each activity [28].

For the HIVST function (offered to participants in the Intervention arm), the cost allocation followed the same rationale used for HCT, but with different proportions. The HCT counsellors were trained to provide a five-minute demonstration on how to use the self-testing kit. Thus, for those who self-tested in the clinic with provider supervision, the total time spent by the counsellor was 35 minutes: (Activities B (5/35), C (25/35) which included the self-test demonstration, and D (5/35). For those who self-tested at home, the total time was 15 minutes, but the activities comprising the self-test at home were still costed out at 35 minutes for methodological consistency: (Activities B (5/35) for the pre-test counselling, C (5/35) for the self-test demonstration, and E (5/35) for the call-back counselling [28].

3. Cost-and outcomes comparison

The costs per client tested were compared across the three RCT arms. In order to calculate the cost per additional client tested in each arm, we divided the number of additional clients tested by the additional cost. This gives an indication of the value for money of offering HIVST to both FSWs and truckers. The lower the additional cost per client, the greater the value for money, with negative values indicating a cost saving.

4. Sensitivity Analysis

The study performed a series of univariate sensitivity analyses on the major cost drivers and key parameters identified. This determined the robustness of the cost analysis and the impact on the resulting average cost per client and cost per additional client tested. These parameters are discussed below:

- Counsellor salaries: The study used an average of KES 39 322.50 within the cost analysis. The questionnaire indicated that Counsellors’ salaries varied from KES 22 030 to KES 55 080 so the sensitivity analysis varied the parameter accordingly.
- HCT Timeframe: This study used the average of 30 minutes per client in the cost analysis, which, according to the questionnaire could take up to 75 minutes per client (although very rarely and highly unlikely). The range of possible values accounted for within the sensitivity analysis allowed for an HCT timeframe of 45 and 60 minutes. The extreme value of 75 minutes was excluded from the sensitivity analysis as this would not have provided an accurate reflection of costs.
- HIVST cost drivers: The cost of the HIVST kit and the supporting costs were subjected to a sensitivity analysis to explore assumptions made by other costing studies as listed below.
  ○ HIVST test kit: The price of the HIVST kit was USD 9.22, however, the price dropped to USD 2.00 as per the Gates foundation agreement which took place post the RCT [32].
  ○ HIVST support component: The staff and all other resource-related costs were eliminated as part of a sensitivity analysis to determine the cost of HIVST based solely on the provision of the kit, without the five-minute demonstration (unsupervised HIVST), as explored by other HIVST costing studies reviewed. This illustrated the difference in cost between a supervised (as costed by this study) and unsupervised HIVST strategy.
- Activity A cost: The supporting costs of Activity A are eliminated in the sensitivity analysis and only the direct costs (service provider and data charges) are included to evaluate the effect on the cost per additional person tested. This assumption is made on two costing studies which used an automated system and software to send text message reminders to clients [23, 33].

Finally, a scenario analysis was conducted that estimated optimal response rates for the SMS and HIVST interventions, and a reduced cost for HIVST kits of USD 5.00 and USD 2.00 (the latter a 2017 negotiated price agreement) [34]. The number of participants who returned for testing was increased according to the literature which suggested a 55% response to the receipt of three informational text messages [23]. The proportion of self-testers was increased to 80% of the participants who returned for testing within the Intervention arm. This assumption was based on average results of two studies that showed 72.97%, 82.7% and 84.2% of study participants preferred HIVST over facility-based testing [16, 35].

### Ethics

The study procedures, including the passive consent process, were approved by the City University of New York Institutional Review Board, the Kenya Medical Research Institute Ethics Committee, and the University of KwaZulu-Natal Biomedical Research Ethics Committee.

## Results

### Costs

Table 2 illustrates the total cost of the RCT by study arm and cohort. The total cost for the FSWs was 55% more that of the truck drivers due to the larger number of FSWs that returned for testing. However, the cost per FSW client tested was substantially lower than the cost per trucker tested across all study arms. The cost per client tested for the truckers ranged between USD 20.92 and USD 33.57, with the lowest cost per client tested attributed to the Intervention, followed by the SOC arm, with the Enhanced SOC having the highest cost per client tested. The cost per client tested for the FSWs ranged between USD 9.56 to USD 11.43, with the lowest cost per client stemming from the SOC arm, followed by the Enhanced SOC, and highest cost per client tested attributable to the Intervention arm.

**Table 2:** Total costs and cost per client by study arm, truck drivers and FSWs (activities A–E).

	Number of HCT Clients	Total Cost per Activity (USD)					Total Cost per Study Arm (USD)	Cost per Client (USD)
		Activity A	Activity B	Activity C	Activity D	Activity E		
**Truck Drivers**								
**Intervention**	26	289.23	17.24	218.68	15.98	2.90	544.03	20.92
**Enhanced SOC**	10	289.02	7.06	32.56	7.06	-	335.69	33.57
**SOC**	10	238.13	7.06	32.56	7.06	-	284.81	28.48
**Total: Study Costs**	46	816.38	31.35	283.81	30.09	2.90	1 164.53	
**% of Total Costs**		70.10%	2.69%	24.37%	2.58%	0.25%	100.00%	
**Female sex workers**								
**Intervention**	81	258.34	54.73	552.02	50.30	10.16	925.55	11.43
**Enhanced SOC**	46	258.34	32.45	149.79	32.45	-	473.04	10.28
**SOC**	43	210.49	30.34	140.02	30.34	-	411.18	9.56
**Total: Study Costs**	170	727.17	117.52	841.82	113.09	10.16	1 809.77	

Note: Activity A: Sending SMSs; Activity B: Pre-test counselling; Activity C: HIV test; Activity D: Post-test counselling; Activity E: Call-back counselling

The costs in Table 2 are broken down by SMS-related cost and HCT/HIVST cost in Tables 3 and 4, respectively. Table 3 illustrates the SMS-related costs of the RCT costed as Activity A.

**Table 3:** Total SMS costs and average SMS cost per client (Activity A).

	Truck Drivers			FSWs		
	Intervention	Enhanced SOC	SOC	Intervention	Enhanced SOC	SOC
**Number of SMSs sent**	8 406	8 400	6 921	8 384	8 384	6 831
**Total cost per SMS (USD)**	0.031	0.031	0.031	0.031	0.031	0.031
**Total SMS cost per study arm (USD)**	258.07	257.88	212.48	258.34	258.34	210.49
**Number of clients tested per arm**	26	10	10	81	46	43
**SMS cost per client tested (USD)**	9.93	25.79	21.25	3.19	5.62	4.90

**Table 4:** Average cost per HCT and HIVST client (activities B, C, D and E).

	Resource	HCT Cost per Client (USD)	HIVST Cost per Client (USD)	
			In clinic	Home use
**Staff**	HCT counsellors	1.15	1.35	0.58
**Consumables/medical supplies/running costs**	Various	0.43	9.37	9.42
**Equipment**	Cell phones			0.62
**Infrastructure**	Clinic site	0.83	0.83	0.36
**Training/recruitment**	Once-off Training	0.03	0.03	0.01
	Once-off recruitment	0.00	0.00	0.00
**Overheads and supervision**	Site co-ordinator	0.51	0.51	0.22
	General overheads	2.05	2.05	0.88
	** **	5.01	14.13	12.08

Total: HIVST Cost per client

Note: Activity B: Pre-test counselling; Activity C: HIV test; Activity D: Post-test counselling; Activity E: Call-back counselling

The total SMS cost for the trucker sample was 21% higher for the Intervention and Enhanced SOC arms, at USD 258.07 and USD 257.88, respectively, compared to USD 212.48 for the SOC arm. This is as a result of fewer SMSs sent to participants in the SOC arm than in the other two arms.

For truckers, the total SMS cost per client tested in the Intervention arm was USD 9.93, whereas the cost per client tested in the Enhanced SOC and SOC arms was substantially higher at USD 25.79 and USD 21.25, respectively. Although the total SMS cost was highest in the Intervention arm, the SMS cost per client tested was significantly lower due to the higher number of clients who tested in that arm. This trend was similar for FSWs with the Intervention and Enhanced SOC arms costing more than the SOC arm; however, cost per client tested was lower, driven primarily by the greater number of FSWs who tested.

The average cost per client tested attributed to HCT and HIVST is broken down by economic classification in Table 4, where HCT refers to provider-administered HIV testing and HIVST refers to self-administered testing.

The total costs of the counselling and testing component of the interventions varied, depending on whether the client opted for the self-test or conventional provider clinic-based test. A further difference in cost was dependent on whether the client chose the in-clinic self-testing option, or the home use self-testing option. The cost per provider-administered HIV test was calculated at USD 5.01, while for HIVST, the cost was more than double, at USD 14.13 and USD 12.08 for in clinic and out of clinic HIVST respectively. This was mostly driven by the cost of the test kit itself (USD 7.95 +16% VAT), included in Table 4 under consumables/medical supplies/running costs. For conventional HCT, the bulk of the cost was attributed to staff costs (USD 1.15) and general clinic overheads (USD 2.05). For HIVST, all costs other than the consumables/medical supplies/running costs and staff costs were identical to or lower than those calculated for HCT.

### Outcomes

The HIV testing outcome data for each of the three study arms are described in Table 5.

**Table 5:** Proportional outcomes by study arm, truck drivers and female sex workers.

	Truckers			Female Sex Workers		
Outcomes	Intervention	Enhanced SOC	SOC	Intervention	Enhanced SOC	SOC
**Number of Participants per Arm**	750	748	762	750	750	696
**Number of Participants: All Clinic Services**	94	80	81	129	70	70
**Percentage of Participants: All Clinic Services**	12.53%	10.70%	10.63%	17.20%	9.33%	10.06%
**Number of Participants Tested**	26	10	10	81	46	43
**HIVST (in clinic)**	13	-	-	26	-	-
**HIVST (home use)**	2			7		
**HCT**	11	-	-	48	-	-
**Percentage of Participants Tested**	3.47%	1.34%	1.31%	10.80%	6.13%	6.17%

The percentage of male truckers who tested was low across the RCT, with 3.47% testing in the intervention arm and 1.34% and 1.31% testing in the Enhanced SOC and SOC arms, respectively. Of the 26 male truck drivers in the Intervention arm who tested, 15 (57.7%) opted to self-test and 11 opting for the provider-administered blood test. The percentage of FSWs who tested during the RCT was higher, with 10.80% testing in the Intervention arm, whilst 6.13% and 6.17% tested in the Enhanced SOC and SOC arms, respectively. Of the 81 FSWs in the Intervention arm who tested, 26 (32.1%) opted to self-test at the facility, 7 to self-test at home, and the remaining 48 opting for the provider-administered HIV test.

### Cost per additional client tested results

The incremental cost per additional client tested in the Enhanced SOC relative to the SOC, and the Intervention relative to the Enhanced SOC, are presented in Table 6 below.

**Table 6:** The cost per additional client tested across study arms for truckers and FSWs.

		Cost per Client (USD)	Number of clients tested	Change in Cost (USD)	Additional number of clients tested	Cost per additional client tested (USD)
**Truckers**	**SOC**	26.26	10			
	**Enhanced SOC**	30.80	10	4.54	0	- (
	**Intervention**	21.48	26	-9.32	16	-0.58
**FSWs**	**SOC**	9.90	43			
	**Enhanced SOC**	10.63	46	0.72	3	0.24
	**Intervention**	15.80	81	5.18	35	0.15

The Intervention arm had the lowest cost per additional client tested for the trucker sample, driven primarily by the increased number of testers at USD -0.58 compared to the enhanced SOC. The same was observed for the FSW cohort, at a cost of USD 0.15 per additional client tested in the Intervention arm.

### Sensitivity analysis

The results of the sensitivity analysis are shown in Table 7.

**Table 7:** Cost sensitivity analysis for trucker cohort.

		Average Salary (USD 387.38)		Minimum Salary (USD 217.02)		Maximum Salary (USD 542.61)	
		Cost per Client (USD)	Cost per additional client tested (USD)	Cost per Client (USD)	Cost per additional client tested (USD)	Cost per Client (USD)	Cost per additional client tested (USD)
**Salary**	**SOC**	26.26		25.75		26.72	
	**Enhanced SOC**	30.80		30.29		31.26	
	**Intervention**	21.48	-0.58	20.99	-0.58	21.93	-0.58
** **	** **	**30 Minutes**		**45 Minutes**		**60 Minutes**	
**Time taken for testing**	**SOC**	26.26		26.83		26.83	
	**Enhanced SOC**	30.80		31.37		31.37	
	**Intervention**	21.48	-0.58	21.61	-0.61	21.82	-0.60
** **	** **	**Test Kit = USD 9.22**		**Test Kit = USD 5.00**		**Test Kit = USD 2.00**	
**Price of HIVST kit**	**SOC**	26.26		26.26		26.26	
	**Enhanced SOC**	30.80		30.80		30.80	
	**Intervention**	21.48	-0.58	18.23	-0.79	15.93	-0.93
	** **	**Supervised**		**Unsupervised**			
**Supervised vs unsupervised testing**	**SOC**	26.26		26.26			
	**Enhanced SOC**	30.80		30.80			
	**Intervention**	21.48	-0.58	19.41	-0.71		
	** **	**Including Supporting Costs**		**Excluding Supporting Costs**			
**Supporting costs**	**SOC**	26.26		17.58			
	**Enhanced SOC**	30.80		20.26			
	**Intervention**	21.48	-0.58	17.43	-0.18		

The sensitivity analysis undertaken on the trucker cohort, revealed that a lower average monthly salary (by 43.98%) reduced the cost per client across all three study arms by approximately USD 0.5, while the cost per additional client tested for the Enhanced SOC and Intervention remained the same. The same effect was observed in reverse when implementing a 40.07% increase in salaries.

Spending an extended period of time with clients (45 minutes) increased the cost per client by USD 0.58 across the SOC and Enhanced SOC study arms, compared to a USD 0.13 increase in the Intervention arm. The cost per additional client tested for the Intervention was lowered by USD 0.03. Doubling the average HCT timeframe to 60 minutes increased the cost per client by USD 0.58, across the SOC and Enhanced SOC, and by USD 0.34 for the Intervention arm. The cost per additional client tested decreased by a further USD 0.01 for the Intervention arm.

Because the cost of an HIVST kit was a key driver in the costs of the intervention, sensitivity analysis on this parameter had a large effect on the intervention costs. Changing the cost to USD 5 decreased the cost per client tested and the cost per additional client tested for the Intervention arm by USD 3.25 and USD 0.20, respectively. Further, decreasing the cost of the HIVST kit to USD 2.00 resulted in a USD 5.56 decrease in the cost per client as well as a USD 0.35 decrease in the additional cost per client tested for the Intervention arm.

Sensitivity analysis of supervised verses non-supervised HIVST revealed a difference in cost. Removing the costs associated with supervision resulted in a decrease of USD 2.07 and USD 0.13 in the cost per client and additional cost per client tested within the Intervention arm, respectively.

Removing the supporting costs (staff time) associated with effecting Activity A (SMS intervention) resulted in a decrease of USD 8.68, USD 10.54 and USD 4.06 in the cost per client in the SOC, Enhanced SOC and Intervention arms, respectively.

### Scenario analysis

Following from the sensitivity analysis, a scenario analysis was undertaken in which the cost per client tested and the resulting cost per additional client tested were considerably lower, using realistic assumptions drawn from the literature reviewed. The assumptions underpinning the results presented in Table 8 are as follows: An HIVST test kit price of USD 2 was assumed (34), along with the existing 80% preference for HIVST in the intervention arms (16, 35). The Enhanced SOC response rate is increased to 55% (23), while that of the Intervention arm increased first to 65%, and then 70% (23). The ratio of in-clinic to at home self-testing was maintained from the actual outcomes of the RCT (7:3). From the results above, the threshold at which the cost per additional client tested is lowest for the Intervention, with all other assumed values constant, lies between a response rate of 65% and 70%.

**Table 8:** Scenario analysis of cost determinants.

	Actual Outcomes		65% Uptake		70% Uptake	
	Cost per Client (USD)	Cost per additional client tested (USD)	Cost per Client (USD)	Cost per additional client tested (USD)	Cost per Client (USD)	Cost per additional client tested (USD)
**SOC**	26.26		5.54		5.54	
**Enhanced SOC**	30.80		5.64	0.01	5.64	0.0145
**Intervention**	21.48	-0.58	7.21	0.04	6.53	0.0075

## Discussion

HIVST is considered a key intervention in achieving the UNAIDS 90-90-90 targets [1, 32], with HIVST postulated as a cost-effective means to increasing the uptake of HIV testing [17, 36]. This is the first study to calculate the cost of providing HIVST in a clinic setting, using SMS technology to raise awareness of the availability of this new HIV testing modality.

The use of SMS technology to alert clients to the availability of HIVST at the North Star Alliance clinics in Kenya resulted in an increase in testing rates amongst both truckers and FSWs. The cost analysis revealed that for the trucker sample, moving from the SOC to the Enhanced SOC incurred an increase in cost of USD4.50, but this was accompanied by no change in outcome. Moving between the Enhanced SOC and the Intervention arm resulted in a cost-saving of USD 0.58 per additional tester, as the cost per client tested in the Intervention arm was lower than in the Enhanced SOC. For FSWs, the costs in the Enhanced SOC arm were higher than those in the SOC arm, and with a slightly higher number of people presenting for testing, with a cost per additional client tested of USD 0.24. Offering HIVST resulted in a far higher number of people presenting for testing, at only a small cost per additional client tested of USD 0.15. Even for FSWs, offering HIVST did not result in a cost-saving; however, the incremental cost per additional client tested is very low compared to the total cost, and likely to be considered good value for money if this modality increases testing among these difficult-to-reach populations. The use of SMSs was only found to be justifiable if the associated increase in outcome was substantial, as demonstrated in the Intervention arm. As there was no benefit observed in the SOC and Enhanced SOC arms from increasing the number of SMS reminders, this study suggests that similar outcomes might have been achieved by sending a once-off SMS announcement about HIVST availability, which would decrease the operational costs of a similar intervention in the future.

The cost of the HIVST kits made the cost per client tested using HIVST far higher than the SOC. However, given the substantially lower price of the HIVST kits that the Kenyan government has negotiated with suppliers, the sensitivity analysis revealed that this negotiated test kit price would reduce the cost of HIVST substantially. The scenario analysis further confirmed that additional cost savings could be realised if in-clinic supervision of HIVST was minimised. In-clinic supervision is likely to be minimised once HIVST is normalised and individuals wanting to adopt this testing modality become familiar and more confident to utilise the HIVST kit independently. Clear and succinct instructions on the HIVST kit will further reduce the demand for supervision from health personnel.

The scenario analysis concluded that an increased response rate of between 65–70% would have resulted in a lower cost per client tested in the intervention arm relative to the Enhanced SOC arm. This analysis, using the reduced HIVST kit price, illustrates the importance of ensuring optimal demand for HIVST in order to realise a cost equilibrium with HCT offered at the NSA clinics in Kenya.

To date, this is the first study to evaluate the costs of using mobile technology and HIVST in combination to increase the uptake of HIV testing among high-risk populations in Kenya. Furthermore, in Sub-Saharan African there is only one other primary cost analysis on the use of HIVST [17, 36] and a single study that refers to the cost of mobile technology to increase the uptake of HCT [23]. This study, therefore, contributes useful data on the costs and outcomes of two strategies aimed at increasing HIV testing among hard-to-reach, high-risk populations in Africa.

### Limitations

Differences between the results of this study and those reviewed include a lower impact on HCT uptake observed in this study, and the resultant higher cost per self-tested client [16, 23, 35]. Reasons for the lower than expected outcomes could include seasonal distortions, as the RCT (targeting truckers) was conducted between December 2016 and February 2017, overlapping the festive season, where truckers were possibly on leave and less likely to access roadside clinics. In addition, assigned routes may have prohibited access to North Star Alliance clinics during the study period. Furthermore, the method of sampling from the EHRS may have been flawed due to possible data errors in the system. The results of this study cannot be generalized to all truckers and FSWs in Kenya, let alone outside of Kenya, as the sample size was selected from among those registered in the North Star Alliance EHRS, which only includes clients who have utilized North Star Alliance clinics previously, and because even among this select group, we further sampled according to specific eligibility criteria. The collection of cost data relied heavily on reports from facility and head office staff rather than analysis of invoices and timing activities; hence, there may be errors in certain cost parameters used within the analysis. This study is limited to analyzing the costs associated with the various testing strategies and therefore does take into account which strategy maximizes the health of the population. Therefore, this study acknowledges the limitation of using the cost per client as an outcome measure. This study used USD 7.99 plus 16% VAT as the cost of the HIVST kit. However, the Bill & Melinda Gates Foundation has recently announced an agreement to lower the price per OraQuick HIV Self-test to USD 2.00 across 50 high-burdened lower and middle-income countries (LMICs) in Africa. Although this analysis suggests that the intervention in the RCT was more successful in recruiting clients for testing, and broadly would be worth the incremental cost to increase testing in these difficult to-to-reach and high-risk groups, this is not a cost-effectiveness analysis. Future investigations should include a cost-effectiveness or cost-utility analysis which evaluates health outcomes rather than simply the number of clients who test. However, given the importance of increasing HIV, cost-effectiveness should not be a primary consideration for policy makers considering rolling out low-cost interventions aimed at increasing HIV testing, especially among hard-to-reach and key populations. Rather, the focus should be on improving delivery mechanisms and technical efficiency of interventions that work.

## Conclusion

This is the first study to cost an intervention which successfully utilised mobile technology in alerting truckers and FSWs to the availability of HIVST. Increasing testing rates is a key factor in reducing the cost per additional client tested for this intervention. Work therefore remains to be done in order to maximize outcomes and reduce identified key cost drivers. This study contributes relevant costing data which can inform future interventions involving mobile technology and HIVST within similar settings.
